# Ultrafast demagnetization in iron: Separating effects by their nonlinearity

**DOI:** 10.1063/1.5040344

**Published:** 2018-08-22

**Authors:** Kevin Bühlmann, Rafael Gort, Gerard Salvatella, Simon Däster, Andreas Fognini, Thomas Bähler, Christian Dornes, C. A. F. Vaz, Andreas Vaterlaus, Yves Acremann

**Affiliations:** 1 Laboratory for Solid State Physics, ETH Zurich, 8093 Zurich, Switzerland; 2 Department of Quantum Nanoscience, TU Delft, 2600 GA Delft, The Netherlands; 3 Institute for Quantum Electronics, ETH Zurich, 8093 Zurich, Switzerland; 4 Swiss Light Source, Paul Scherrer Institut, PSI, 5232 Villigen, Switzerland

## Abstract

The laser-driven ultrafast demagnetization effect is one of the long-standing problems in solid-state physics. The time scale is given not only by the transfer of energy, but also by the transport of angular momentum away from the spin system. Through a double-pulse experiment resembling two-dimensional spectroscopy, we separate the different pathways by their nonlinear properties. We find (a) that the loss of magnetization within 400 fs is not affected by the previous excitations (linear process), and (b) we observe a picosecond demagnetization contribution that is strongly affected by the previous excitations. Our experimental approach is useful not only for studying femtosecond spin dynamics, but can also be adapted to other problems in solid-state dynamics.

## INTRODUCTION

In 1996, Beaurepaire *et al.*^1^ discovered that demagnetization processes can occur within less than a picosecond, which is below the time responses typically associated with spin precession. In their paper, the three-temperature model (3TM) was introduced, where the couplings between the spin system, the electron gas, and the lattice are described in terms of energy transfer. Following their work, the mechanism responsible for femtosecond angular momentum transfer has been the focus of ultrafast spin dynamics research.

The laser-induced ultrafast loss of spin angular momentum can be attributed to two effects. (a) The electron spins can flip in the excited ferromagnet due to collisions with phonons^2^ and hot electrons^3–5^ by the Elliot-Yafet scattering mechanism.^6^ Spin flip scattering in the bulk is fundamentally caused by spin-orbit coupling.^7–14^ In addition, the temperature-dependent shifts of the chemical potentials for minority and majority electrons have been identified as a driving force for spin flips.^15^ (b) Spin transport, whereby spin-polarized electrons are transported from the magnetic surface deeper into the sample, where they can flip their spin outside the region being probed.^16–18^ This mechanism has been observed experimentally.^19–23^ The spin current can even be injected into a second ferromagnet, where it affects the magnetization through the spin torque effect.^22–26^ However, the spin current alone cannot fully explain ultrafast demagnetization, as shown by Wieczorek *et al.*^27^

Here, we present a dual pump-probe experiment that aims to investigate the nonlinear aspects of the ultrafast demagnetization effect. We use a first pump pulse *P*_h_ to heat the ferromagnet. This pulse arrives at time *τ* before time zero. Its energy is primarily absorbed by the electron gas, leading to an increase in its temperature. Within the electron-lattice equilibration time *τ_el_* ≈ 1.2 ps (determined from the reflectivity signal according to Refs. 28 and 29), the electron gas equilibrates with the lattice to a common temperature. A second pump pulse *P*_d_ excites the sample at time zero. We observe the demagnetization caused by the second pump pulse. Here, we study how the previous excitations from the first pump pulse affects the demagnetization caused by the second pump pulse.

## EXPERIMENTAL SETUP

The sample consists of a single-crystalline Fe layer grown on a substrate of MgO (001) by molecular beam epitaxy. The Fe layer is 17 nm thick, and it is capped by 2 nm MgO and 2 nm Al. The sample is placed inside a cryostat, which allows for cooling down to 10 K, to suppress excitations in the phononic, electronic, and spin system. The average temperature increases to 100 K once the pump laser beams are present. A static magnetic field of 350 Oe is applied, which saturates the magnetization along the easy axis, indicated by ↑, ↓. An amplified Ti:sapphire laser system with a repetition rate of 10 kHz and a pulse length of 25 fs FWHM is used to excite and detect the magnetization. The pump laser is split into heating and demagnetizing pulses, which are delayed independently. The pump section of the experiment was designed to provide equal dispersion for both pulses. In addition, separate compressors are used for the pump and probe pulses, which are optimized for the shortest pulses on the sample. The probe beam is converted from 800 nm to 400 nm using a beta barium borate (BBO) crystal to avoid state blocking effects.^30,31^ We use the longitudinal magneto-optical Kerr rotation to detect the magnetization. The pump beam is modulated by a mechanical chopper at 83 Hz for lock-in detection of the pump-induced demagnetization. The signal is measured for the two magnetization directions ↑ and ↓, and the difference is calculated. This difference represents the laser-induced demagnetization of the sample Δ*M*.

## EXPERIMENTAL RESULTS AND DISCUSSION

**FIG. 1.**
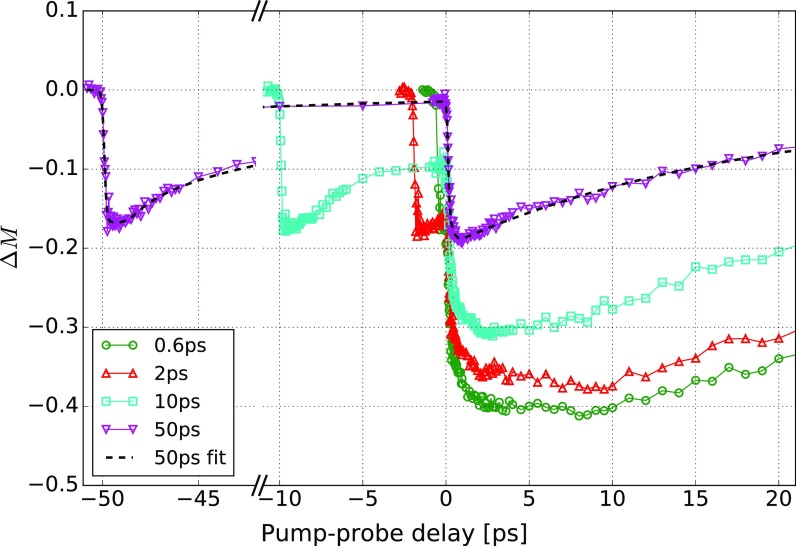
Measured demagnetization Δ*M* caused by a pair of pump pulses (heating pulse *P*_h_ followed by the demagnetization pulse *P*_d_). The excitations caused by *P*_h_ enhance the demagnetization of *P*_d_. The legend indicates the time interval between the heating and demagnetization pulses, *τ*. The line for *τ* = 50 ps shows the fit Δ*M*_fit_ used to calculate Δ*M_d_* shown in Fig. 2.

The demagnetization curves for different pump-pump delays *τ* are shown in Fig. 1. The fluences of both pump pulses are adjusted such that each of them alone demagnetizes the sample by 17%. This is apparent at *τ* = 50 ps. Here, the heating pulse *P*_h_ causes approximately the same demagnetization as *P*_d_. The time of 50 ps is sufficient to cause almost a complete recovery of the magnetization and significant cooling of the electron gas and lattice. However, if *τ* is reduced, then the heating pulse starts to enhance the demagnetization caused by *P*_d_. In addition, for *τ* < 2 ps, the largest demagnetization is not reached after the ultrafast drop near *t *=* *0 but approximately 10 ps later.

To study the temporal behavior of Δ*M*, we subtract the background of the magnetization recovery caused by the heating pulse *P*_h_. The demagnetization of a single pulse is fitted by the empirically determined function

$$\begin{array}{c} \Delta M_{\text{fit}}=\frac{a_{0}}{2}\left(1+\tanh\left(\frac{t-t_{0}}{\tau_{0}}\right)\right)+\Theta (t-t_{1})a_{1}\left(e^{\frac{-(t-t_{1})}{\tau_{1}}}-1\right)+\Theta (t-t_{2})a_{2}\left(e^{\frac{-(t-t_{2})}{\tau_{2}}}-1\right). \end{array}$$ (1)

Here, Θ(*x*) is the Heaviside function. The first term fits the fast decay, and the second and third terms fit the recovery. This single-pulse fit is determined using the first part of the trace at *τ* = 50 ps (before the second pulse hits) and is displayed in Fig. 1. Δ*M*_fit_ is shifted in time and subtracted from each measured demagnetization curve Δ*M*

$$\Delta M_{d}=\Delta M-\Delta M_{\text{fit}}.$$ (2)

The resulting function Δ*M_d_* would be equal to Δ*M* caused by *P*_d_ alone if the magnetization reacted in a linear manner to the pump pulses. For small demagnetization amplitudes, this is actually the case, as demonstrated in Ref. 32. In contrast, we work with larger demagnetization amplitudes of 17% per pulse, driving the system into a nonlinear response regime.

**FIG. 2.**
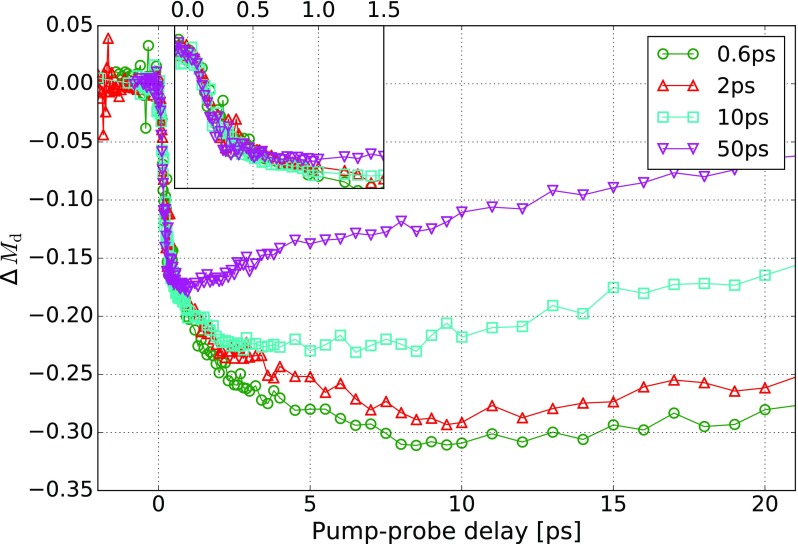
Demagnetization Δ*M_d_* caused by *P*_d_ after subtracting the demagnetization caused by the heating pulse *P*_h_. The inset shows that the ultrafast part of the demagnetization is not affected by the heating pulse. However, on the picosecond time scale, we observe an enhancement in the demagnetization caused by *P*_h_. In the case of a pump-pump delay of *τ* = 0.6 ps, the maximum amplitude of Δ*M_d_* is reached at *t *≈* *10 ps.

The result is shown in Fig. 2. We distinguish between the initial, ultrafast part of the demagnetization at *t *<* *400 fs and the dynamics occurring on a longer time scale of up to 10 ps. We do not observe a significant effect of the heating pulse *P*_h_ on Δ*M_d_* for the ultrafast part of the demagnetization (visible in the inset of Fig. 2). All the measurements of Δ*M_d_* for *t *<* *400 fs are equal within the margin of error and are independent of the pump-pump delay time *τ*. The ultrafast demagnetization process is therefore linear within the accuracy of our experiment. This linear effect is not predicted by the magnetic three-temperature model.^2^

In contrast, the response for *t *>* *1 ps strongly depends on *τ* and is therefore affected by the heating pulse, as shown in Fig. 2. The demagnetization is enhanced by the heating pulse *P*_h_, and it reaches its maximum up to 10 ps after the demagnetizing pulse at *t *=* *0 (for *τ* < 2 ps).

The following question thus arises: which of the reservoirs (spin system, electron gas, or the lattice) excited by *P*_h_ causes the enhancement of the demagnetization? Here, we define the *enhancement* Δ*M*_e_ as the maximum deviation between the demagnetization with and without the heating pulse *P*_h_

$$\Delta M_{e}(\tau )=-\max_{t}\left(\left|\Delta M_{d}(\tau ,t)|-|\Delta M_{\text{no}\;\text{heating}}(t)\right|\right).$$ (3)

**FIG. 3.**
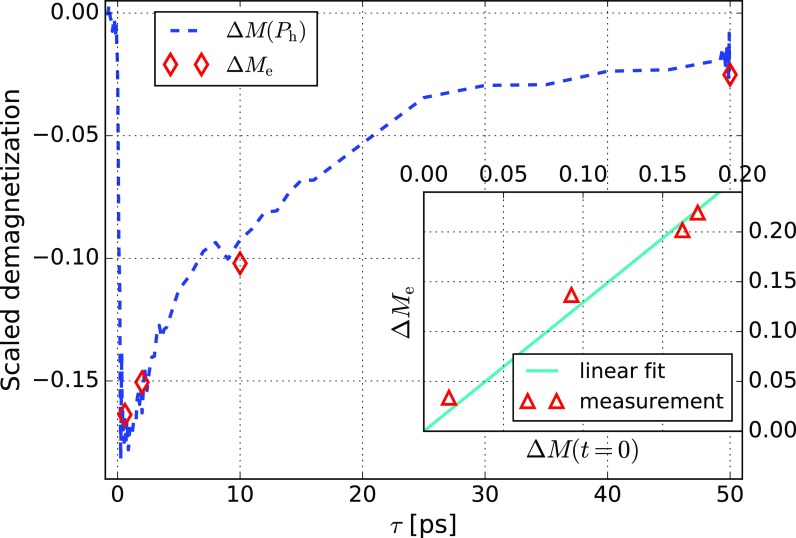
Correlation between the enhancement Δ*M_e_*(*τ*) (diamonds) and the demagnetization caused by a single pump (dashed line) Δ*M*(*P_h_*). Note that Δ*M*_e_ has been scaled in amplitude to match Δ*M*(*P_h_*). The inset shows the linear relation between the measured demagnetization just before *P*_d_ is applied [given as Δ*M*(*t *=* *0, *τ*)] and Δ*M*_e_.

In order to keep the average heat load on the sample as well as the average temperature constant, the demagnetization without heating pulse has been determined by shifting the heating pulse to a time after the measurement pulse. The enhancement Δ*M_e_* is plotted as a function of *τ* in Fig. 3. The strongest enhancement is observed for *τ* = 0.6 ps at *t *=* *7 ps.

We find a correlation between the enhancement Δ*M_e_* and the demagnetization just before *P*_d_: Fig. 3 shows a linear relation between the two quantities. This result suggests that magnetic excitations may be the source of the enhancement Δ*M*_e_. This is consistent with the model proposed by Mueller *et al.*,^15^ where they identify the magnetization-dependent shift of the exchange splitting as a feedback mechanism causing a larger separation of the spin-split chemical potentials and therefore more spin flips.

Cheng *et al.*^33^ performed a similar double-pump - probe experiment on TbFeCo to investigate the nonlinear effects of the demagnetization process. Our results agree with their atomistic Landau-Lifshitz-Gilbert model, which shows that excitations of the spin system can lead to further demagnetization.

## CONCLUSIONS

We conclude from our results that the ultrafast loss of the magnetization within the first 400 fs is not enhanced by the heating pulse. Thus, it is not affected by the previous generation of hot electrons, phonons, or magnons within the margin of error of our experiment. A possible mechanism relevant on this time scale is the spin transport effect. However, we observe a significant (nonlinear) enhancement in the demagnetization on a longer time scale of up to 10 ps. The enhancement is proportional to the demagnetization caused by the first pump pulse, which indicates that the presence of disorder in the spin system enhances the spin flip probability.

Our results indicate that a new framework is needed to fully understand the observed ultrafast demagnetization phenomena, including the fully linear ultrafast contribution. The experimental results indicate that the mechanism for the ultrafast loss of the magnetization is different from the mechanism causing the slow drop on the picosecond time scale.^29^ We suggest that novel spin- and time-resolved photoemission experiments will be able to separate and identify the two effects^34,35^ and shed further light on the fundamental processes underlying the mechanism for ultrafast demagnetization.

Furthermore, our experiment shows that the nonlinear aspects of ultrafast processes in solids can be used to separate similar ultrafast contributions. Such an approach could be useful for other problems in condensed matter dynamics, particularly in the case of correlated systems.
